# Maternal determination of soldier proportion and paternal determination of soldier sex ratio in hybrid *Reticulitermes* (Isoptera: Rhinotermitidae) termite colonies

**DOI:** 10.1371/journal.pone.0293096

**Published:** 2023-11-02

**Authors:** Yao Wu, Jiaming Chen, Mamoru Takata, Kenji Matsuura

**Affiliations:** Laboratory of Insect Ecology, Graduate School of Agriculture, Kyoto University, Kyoto, Japan; Ain Shams University Faculty of Agriculture, EGYPT

## Abstract

Altruistic caste, including worker and soldier (derived from worker), plays a critical role in the ecological success of social insects. The proportion of soldiers, soldier sex ratios, and the number of workers vary significantly between species, and also within species, depending on colony developmental stage and environmental factors. However, it is unknown whether there are sex-linked effects from parents on controlling the caste fate or not. Here, we compared soldier sex ratios, soldier proportions, and population size among a four mating types of *Reticulitermes amamianus* (Ra) and *R*. *speratus* (Rs) (male × female, mRa × fRa, mRa × fRs, mRs × fRa, mRs × fRs) and demonstrate that the soldier sex ratio and worker population size of hybrid colonies skew to colonies of king’s species, while the soldier proportion skew to queen’s species. The survival rate of offspring resulting from interspecies hybridization was significantly higher for mRa × fRs than for mRs × fRa. The results of this study demonstrate the asymmetric influence of kings and queens on caste determination and colony growth, which can contribute to our better understanding of parental influence on the colony dynamics of social insects.

## Introduction

In the ecological and evolutionary progression of social insects, division of labor plays an integral role, as progeny differentiate into either reproductive (nymph) or functionally sterile castes [1–3]. Workers are primarily involved in tasks like foraging, tunneling, tending to eggs, and maintaining hygiene, whereas colony defense is predominantly the role of the soldiers, and reproduction is solely performed by the king and queen [4–6]. As reviewed by Schwander [7], various environmental factors such as nutritional content and/or abundance, temperature conditions, queen pheromones, queen age and/or hibernation state, as well as colony size, have been demonstrated to influence the production of reproductives. However, the accumulation of extensive data over the past two decades has decisively refuted the once-prevailing notion that broods of social insects possess complete totipotency, and that environmental factors are the exclusive determinants of caste allocation, as comprehensively reviewed by Matsuura [8]. It is demonstrated that the caste of offspring is influenced by the sex-antagonistic action of epigenetic factors inherited from the king and queen in termites [9]. Thus, parental effects encompassing transgenerational epigenetic components or behavioral factors, such as parent-to-offspring proctodeal trophallaxis, can play a crucial role in determining the caste fate of the offspring [10–12].

**Fig 1:**
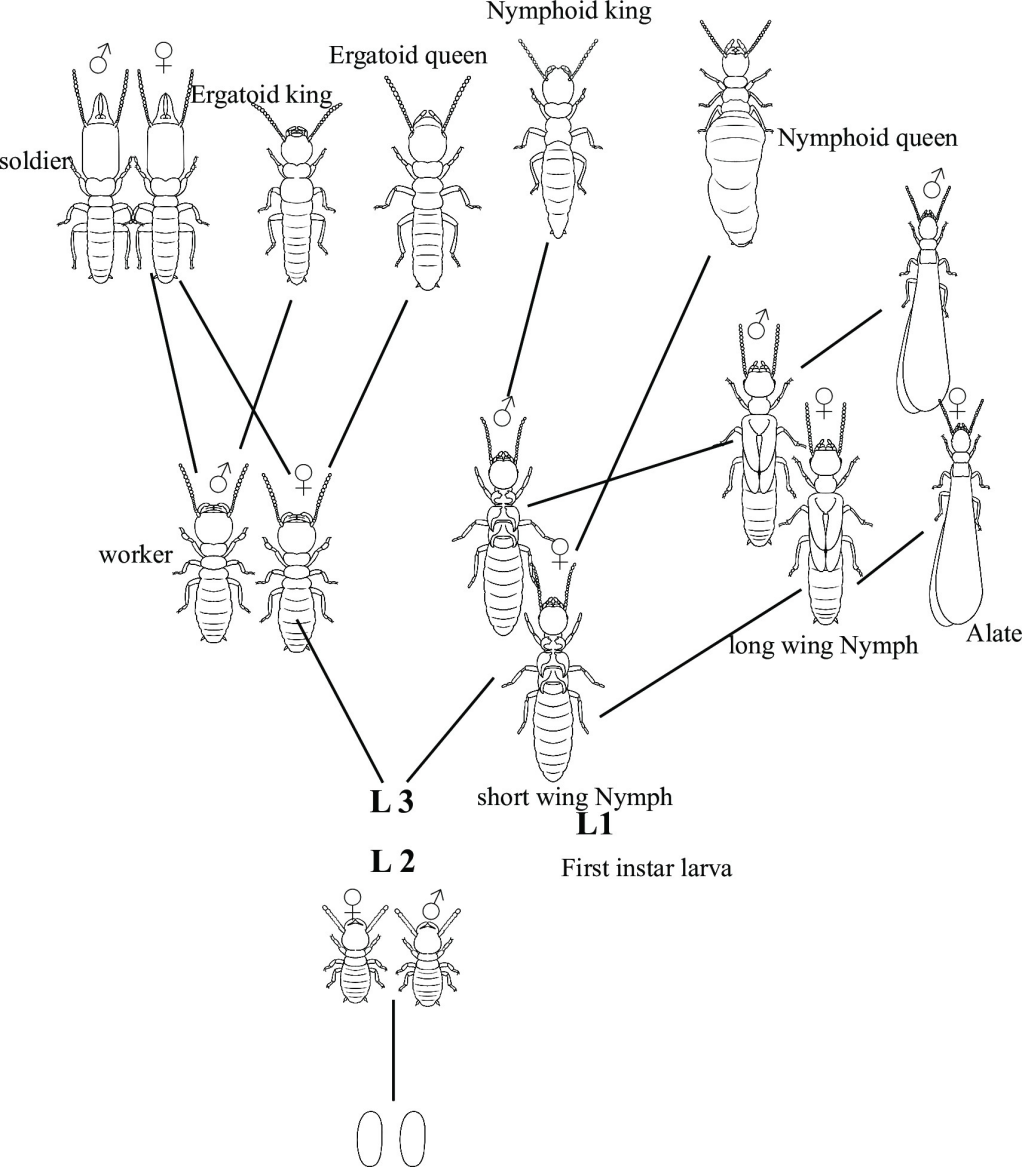
Caste developmental pathways in the *Reticulitermes* termites. The larvae differentiate into either nymphs or workers. Nymphs eventually molt into alates and fly away to establish new colonies.

In this paper, we investigated the sex-linked parental effects on the caste fate by using laboratory hybrid colonies of *Reticulitermes* termites. Accumulating evidence suggests that hybrid termite colonies can inherit phenotypic traits from both parental species [13–15]. *Reticulitermes* soldiers differentiate from fourth- or fifth-instar workers in mature colonies (Fig 1). The proportion of soldiers within the nest is strategically regulated, as an excessive or inadequate number of soldiers can adversely impact colony performance [16]. According to Matsuura’s theory, sexual size dimorphism (SSD) often associates with a sex ratio bias in soldiers [17]. This is because larger workers are more likely to molt into soldiers, and in some termite species, females are larger than males [17, 18]. Consequently, species with a biased soldier sex ratio might recruit suitable phragmotic soldiers, thereby improving their defensive capabilities. For instance, *R*.*speratus*, *R*. *virginicus*, *R*. *flavipes*, and *R*. *kanmonensis* differ in soldier sex ratio: *R*. *speratus* soldier is biased to females, feminized in *R*. *virginicus*, while it’s equal in *R*. *flavipes* and *R*. *kanmonensis* [17].

In this study, we investigated sex-linked effects from parents on the colony characteristics by using hybrid colonies of *R*. *amamianus* (Ra) and *R*. *speratus* (Rs). First, we collected colonies of these two species in the field and compared the sex ratio of soldiers in mature field colonies, finding that the soldier sex ratio of *R*. *speratus* colonies was skewed towards females, while that of *R*. *amamianus* colonies was skewed towards males. Thus, we compared soldier sex ratio, soldier proportion, and number of workers among four combinations of parent species: mRa × fRa, mRa × fRs, mRs × fRa, and mRs × fRs (m: male, f: female). We performed two rounds of observations and data collection: first in colonies aged two years, and second in the colonies aged two and a half years.

## Materials and methods

### Termite sampling

Decayed logs containing *R*. *amamianus* or *R*. *speratus* colonies were collected in pine or Japanese cedar forests in Kagoshima, Hyogo, Kyoto, Shiga, and Hokkaido, Japan, from 2019–2021 (see S1 Dataset for details). Twenty and 17 colonies of *R*. *amamianus* and *R*. *speratus*, respectively, were used to investigate soldier sex ratio. Additionally, two colonies containing alates for each species were collected in Kagoshima and Kyoto, Japan in 2020, and used in hybrid experiments. Each colony was individually processed in the following experiments.

### Soldier sex ratio in field colonies of *R*. *amamianus* and *R*. *speratus*

All soldiers were extracted from the logs, then up to 100 individuals were randomly selected from each colony. If the total soldier number was less than 100 in the sampled log, all soldiers were used. The soldiers were sexed by sternite morphology [19], and the number of each sex was recorded.

### Foundation of hybrid colonies

All alates were extracted from the logs, had their wings removed, and were sexed by sternite morphology [19]. Then, 720 individuals for each sex and colony were randomly selected and assigned to one of the following crosses: mRa × fRa (mRaC × fRaD, mRaD × fRaC), mRa × fRs (mRaC × fRsA, mRaD × fRsA, mRaC × fRsB, and mRaD × fRsB), mRs × fRa (mRsB × fRaC, mRsA × fRaD, mRsB × fRaD, mRsA × fRaC), and mRs × fRs (mRsA × fRsB, mRsB × fRsA). One hundred and twenty pairs were set for each (see S1 Dataset for details). Each pair was placed in a dish (34 mm diameter × 10 mm height) with brown-rotted pinewood mixed cellulose (BPC) medium [20] for six months, and transferred to a larger plastic box (100 × 65 × 28 mm) filled with the BPC medium and a soil block (36 × 36 × 14 mm, W × D × H). They were maintained at 25°C under dark conditions. We replenished the BPC medium whenever one-third was consumed to ensure a constant supply of food. Two years after the founding of the colony, we transferred the colonies to even larger plastic boxes (194 × 104 × 26 mm, W × D × H) containing two soil blocks and two pieces of pine woods (45 × 45 × 10 mm, W × D × H).

At two and two and a half years after colony foundation, all individuals from each colony were extracted. The numbers of eggs, larvae, workers, and nymphs were recorded. For soldiers, they were sexed by sternite morphology and the counts for each sex was recorded.

### Egg-fostering experiment

To investigate the sex-link parental effects on the offspring survival, we conducted an egg-fostering experiment and compared survival rate among combinations of parent species. We performed three replications in each combination of parent species (i.e., in mRs × fRs colonies, three colonies from each of mRsA × fRsB and mRsB × fRsA crosses were used for egg collection). Twenty eggs were randomly extracted from each colony and transferred into a dish (34 mm diameter × 10 mm height) with BPC medium and 50 male workers. The male workers were selected from a different colony of the same cross.The hatching period in *R*. *speratus* is approximately 35 days [21], so we recorded the number of larvae in each dish every week for 10 weeks in total. The workers who tend to differentiate into neotenic kings or soldiers were removed as soon as possible. If the offspring develop into N1s or W1s, the numbers of individuals of each caste and sex were recorded. The caste and sex were identified by the presence of wing buds and sternite morphology, respectively [19, 22, 23].

### Statistical analysis

Exact binomial tests were applied to compare the observed numerical soldier sex ratio in field-collected colonies in *R*. *amamianus* and *R*. *speratus* against the null hypothesis assuming that the numbers of males and females were equal. The total number of male and female soldiers in each species were used for the analyses.

Comparison of the numerical soldier sex ratio, soldier ratio, offspring survival rate, or offspring sex ratio among four combinations of parent species (mRa × fRa, mRa × fRs, mRs × fRa, and mRs × fRs) was performed with generalized linear models (GLMMs) with a binomial distribution. In the models, the objective variable was the numerical sex ratio of soldiers (number of males vs females), soldier ratio (number of soldiers vs workers), offspring survival rate (number of surviving vs dead larvae), or offspring sex ratio (number of males vs females), the explanatory variable was the combination of parent species, and colony ID was treated as a random factor. Comparison of the number of workers among the four combinations of parent species was performed with a GLMM with Poisson distribution. In the model, the objective variable was the number of workers, the explanatory variable was the combination of parent species, and colony ID was treated as a random factor. We also performed GLMMs which included a species of king × queen interaction term instead of the combination of parent species to account for sex and species-specific effect. Likelihood ratio tests (LRTs) were used to determine the statistical significance of each explanatory variable.

Exact binomial tests were applied to compare the observed numerical offspring sex ratio in each combination of parent species against the null hypothesis assuming that the numbers of males and females were equal. The total number of male and female offspring in each combination of parent species were used for the analyses.

All analyses were performed by the software R v4.2.3 [24], with the ‘lme4’ and ‘car’ packages. For the GLMMs and GLMs, Tukey’s HSD tests using the ‘glht’ function in the ‘multcomp’ package [25] were conducted to test for differences among the combinations of parent species. A significance value of *p* < 0.05 was considered to indicate statistical significance.

## Results

### (a) Comparison of soldier sex ratio

**Fig 2:**
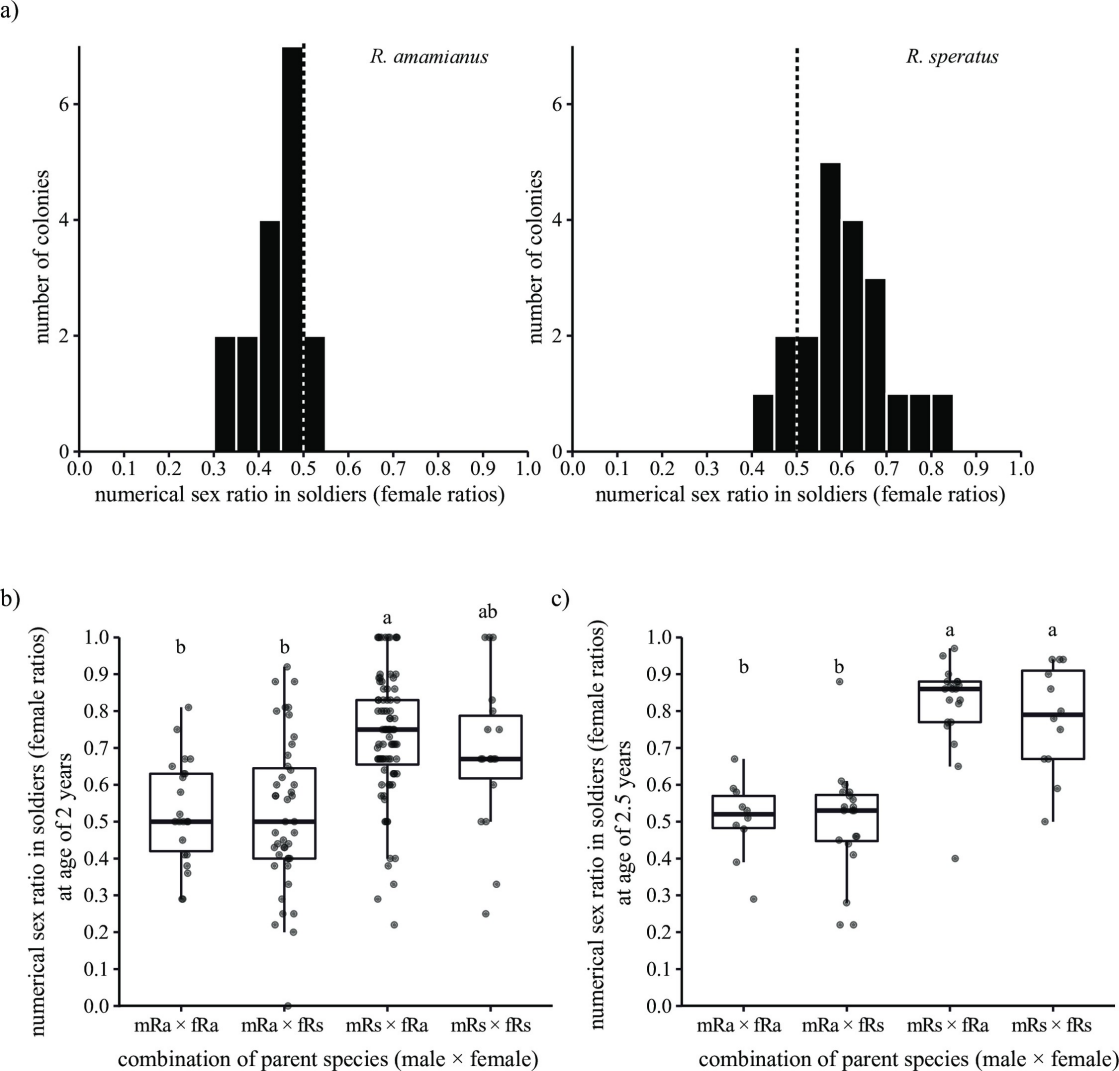
Comparison of sex ratio in soldiers in *R*. *amamianus*, *R*. *speratus*, and their hybrid colonies. a) Biased soldier sex ratio in *R*. *amamianus* and *R*. *speratus* in the field. The dashed line indicates an equal sex ratio. b) and c) Soldier sex ratio in 2- and 2.5-year-old colonies, respectively. The combination of parent species (male × female) shows the species of primary king and queen (i.e., mRa × fRs means primary king is *R*. *amamianus*, primary queen is *R*. *speratus*). Plots indicate the female sex ratio in each colony. Different letters indicate significant differences (Tukey’s HSD tests, *p* < 0.05).

In the field collected *R*. *amamianus* colonies, the mean numerical sex ratio of soldiers (proportion of females) was significantly skewed toward males (exact binomial test, 95% CI = 0.409–0.466, *p* < 0.001, Fig 2A), while in *R*. *speratus* it was significantly skewed toward females (exact binomial test, 95% CI = 0.590–0.634, *p* < 0.001).

The hybrid experiment showed that the species of king had a significant effect on the soldier sex ratio (Tukey’s HSD tests, *p* < 0.05, Fig 2B and 2C), while the species of queen had no significant effect on it. There was no significant interaction between the species of king and queen (2-year-old colonies: GLMM, LRT: *χ*^*2*^ = 0.728, *df* = 1, *p* = 0.394; 2.5-year-old colonies: GLMM, LRT: *χ*^*2*^ = 0.435, *df* = 1, *p* = 0.510).

### (b) Comparison of soldier proportions

**Fig 3:**
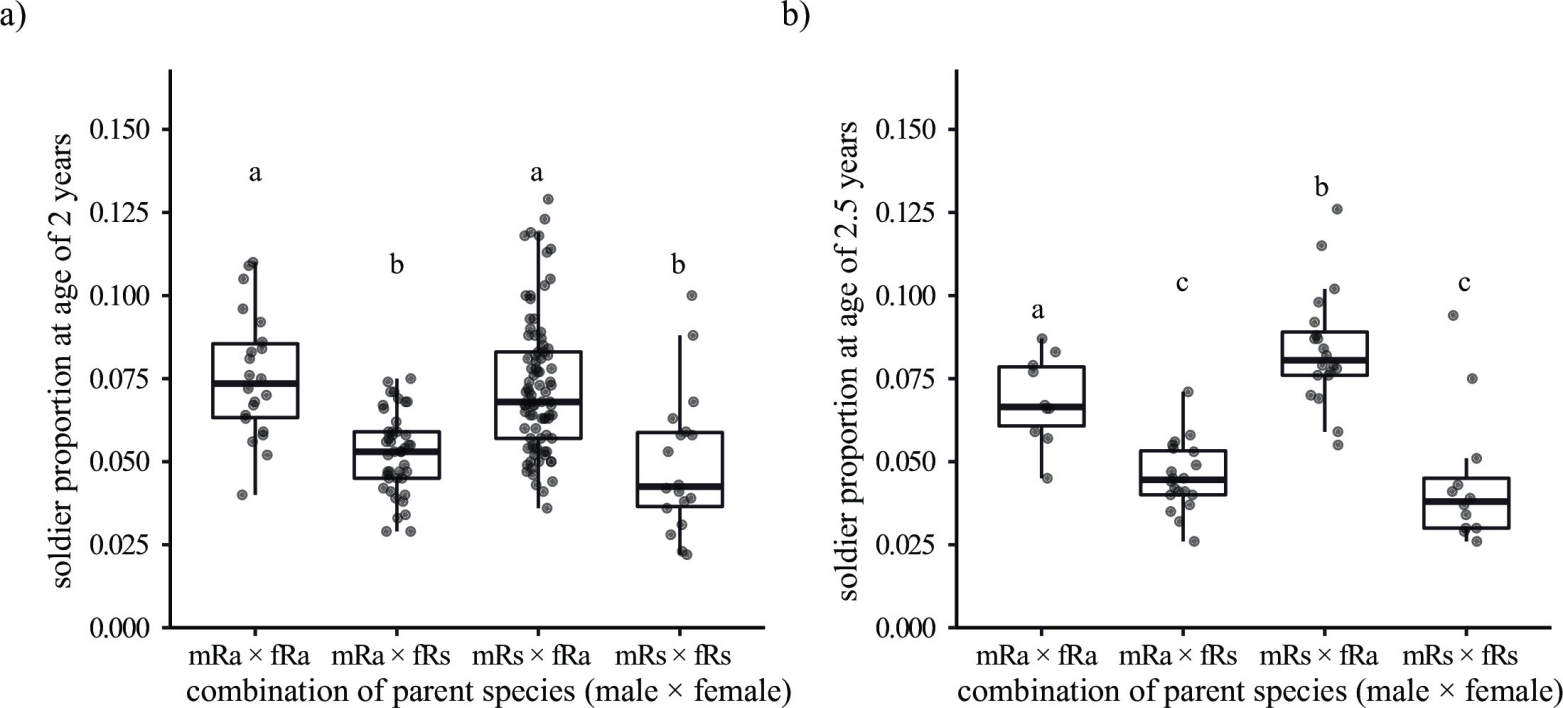
Comparison of soldier proportions in *R*. *amamianus*, *R*. *speratus*, and their hybrid colonies. a) and b) Soldier proportions in 2- and 2.5-year-old colonies, respectively. The combination of parent species (male × female) shows the species of primary king and queen (i.e., mRa × fRs means primary king is *R*. *amamianus*, primary queen is *R*. *speratus*). Plots indicate the soldier proportions in each colony. Different letters indicate significant differences (Tukey’s HSD tests, *p* < 0.05).

The species of queen had a significant effect on the soldier proportions in 2-year-old colonies (Tukey’s HSD tests, *p* < 0.05, Fig 3A), while the species of king had no significant effect. There was no significant interaction between the species of king and queen (GLMM, LRT: *χ*^*2*^ = 1.085, *df* = 1, *p* = 0.298).

The pattern was similar in 2.5-year-old colonies, but there was a significant interaction effect between the species of king and queen (GLMM, LRT: *χ*^*2*^ = 8.058, *df* = 1, *p* = 0.005, Fig 3B). The species of queen had a significant effect on the soldier proportions irrespective of the species of king (Tukey’s HSD tests, *p* < 0.05). In colonies where the queen is *R*. *amamianus*, the species of king had a significant effect (Tukey’s HSD tests, *p* < 0.05), while in colonies where the queen is *R*. *speratus*, the effect was not significant.

### (c) Comparison of number of workers in a colony

**Fig 4:**
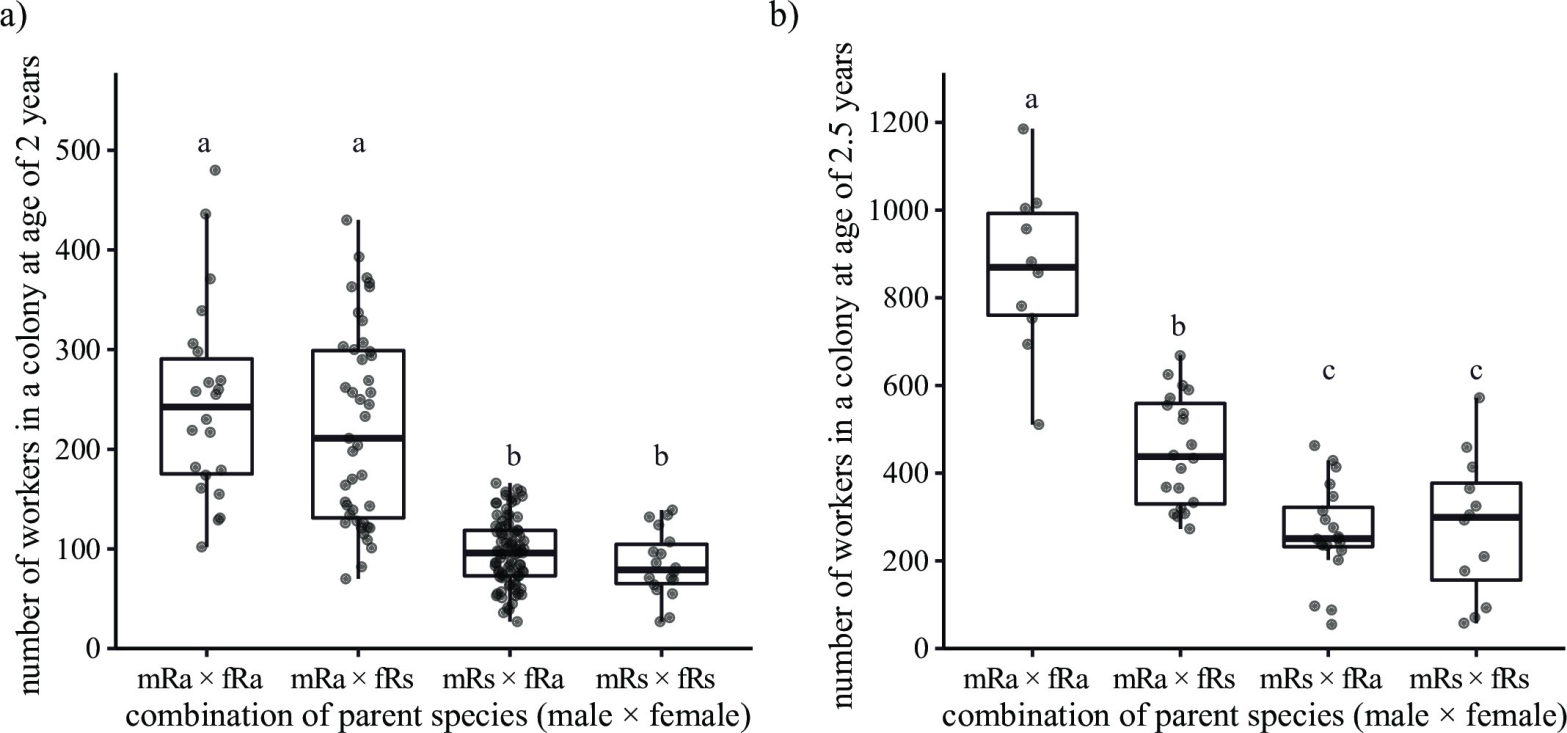
Comparison of number of workers in *R*. *amamianus*, *R*. *speratus*, and their hybrid colonies. a) and b) Number of workers in 2- and 2.5-year-old colonies, respectively. The combination of parent species (male × female) shows the species of primary king and queen (i.e., mRa × fRs means primary king is *R*. *amamianus*, primary queen is *R*. *speratus*). Plots indicate the number of workers in each colony. Different letters indicate significant differences (Tukey’s HSD tests, *p* < 0.05).

The species of king had a significant effect on the number of workers in 2-year-old colonies (Tukey’s HSD tests, *p* < 0.05, Fig 4A), while the species of queen had no significant effect. There was no significant interaction between the species of king and queen (GLMM, *χ*^*2*^ = 0.032, *df* = 1, *p* = 0.859).

In 2.5-year-old colonies, there was a significant interaction between the species of king and queen (GLMM, LRT: *χ*^*2*^ = 6.031, *df* = 1, *p* = 0.141, Fig 4B). The species of king had a significant effect on the number of workers irrespective of the species of queen (Tukey’s HSD tests, *p* < 0.05). In colonies where the king is *R*. *amamianus*, the species of queen had a significant effect on the proportion of soldiers (Tukey’s HSD tests, *p* < 0.05), while in colonies where the king is *R*. *speratus*, the effect was not significant.

### (d) Comparison of offspring survival in egg-fostering experiment

**Fig 5:**
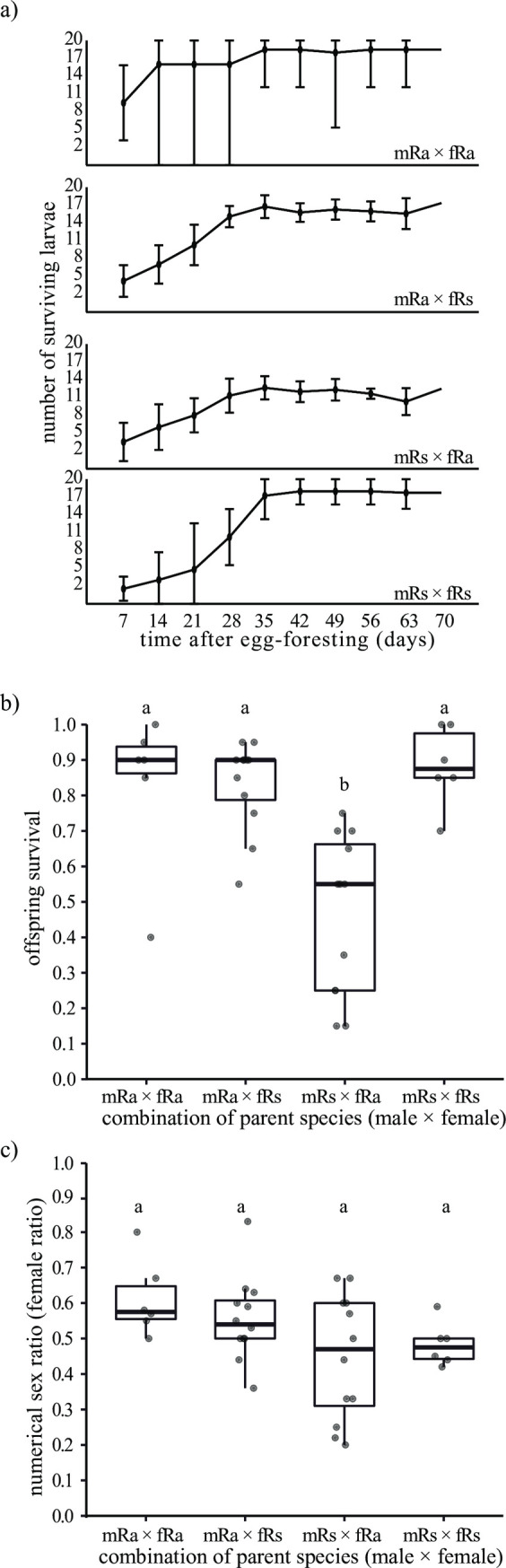
Comparison of offspring survival and sex ratio in *R*. *amamianus*, *R*. *speratus*, and their hybrid colonies. a) Dynamics of number of hatched and surviving larvae in each combination of parent species. Plots and bars indicate mean and 95% confidence interval, respectively. b) and c) Offspring survival rate and sex ratio at the 35th day after egg-fostering, respectively. The combination of parent species (male × female) shows the species of primary king and queen (i.e., mRa × fRs means primary king is *R*. *amamianus*, primary queen is *R*. *speratus*). Plots indicate the number of workers in each colony. Different letters indicate significant differences (Tukey’s HSD tests, *p* < 0.05).

The number of larvae had reached a plateau 35 days after the start of the experiment (Fig 5A). The number of surviving larvae at 35th day were used for comparison of survival rate. There was no significant difference among mRa × fRa, mRa × fRs, and mRs × fRs colonies in the survival rate of fostered offspring except for mRs × fRa combination (Tukey’s HSD tests, *p* < 0.05, Fig 5B).

There was no significant difference in worker sex ratio among the colony types (Tukey’s HSD tests, *p* > 0.05, Fig 5C). The exact binomial tests for each species showed that the larval sex ratio in mRa × fRa was significantly skewed toward females (exact binomial test, 95% CI = 0.503–0.712, *p* = 0.045), while there was no statistically significant bias in the larval sex ratio in mRa × fRs (exact binomial test, 95% CI = 0.466–0.619, *p* = 0.287), mRs × fRa (exact binomial test, 95% CI = 0.346–0.571, *p* = 0.505), and mRs × fRs (exact binomial test, 95% CI = 0.382–0.588, *p* = 0.839). All the offspring were differentiated into workers, and no nymphs were found.

## Discussion

This study unveils the sex-linked parental effects on the caste determination in *Reticulitermes* termites, that is, the king mainly affects the soldier sex ratio and the number of workers, and the queen mainly affects soldier proportion. The closely related subterranean termites *R*. *amamianus* and *R*. *speratus* exhibited distinctions in caste ratios, sex allocation, and population size. Field colonies of *R*. *speratus* displayed a soldier sex ratio skewed towards females, whereas those of *R*. *amamianus* demonstrated a slight male-biased soldier sex ratio (Fig 2A). Incipient colonies of *R*. *speratus* similarly exhibited a female-biased soldier sex ratio, while those of *R*. *amamianus* displayed an equal soldier sex ratio. In *R*. *speratus*, the bias in soldier sex ratio increases as the colony size grows larger (S1 Fig). As shown in Fig 1A, the bias in the soldier sex ratio in *R*. *amamianus* is smaller than that in *R*. *speratus*, even in mature field colonies. Therefore, the absence of a bias in the soldier sex ratio in incipient colonies of *R*. *amamianus* may be attributed to the fact that these colonies had not yet reached a sufficient size to exhibit such a bias. Determining the ratio of soldiers to the total number of individuals within *Reticulitermes* nests proved challenging due to the concealed or formless nature of their nest structures, coupled with the difficulty of defining colony boundaries [26]. Further complicating the matter, environmental factors and colony age significantly influenced the proportion of soldiers [27]. However, it is impossible to identify the environment experienced by the colonies and their age in the field. In this study, by using the laboratory founded colonies, we found that *R*. *amamianus* has larger initial colony sizes (Fig 4) and a higher proportion of soldiers compared to *R*. *speratus* (Fig 3). Investigation of hybrid colonies demonstrate that the soldier sex ratio is consistent with the king’s species exclusively (Fig 2B and 2C); while soldier proportion is consistent with the queen’s species exclusively (Fig 3). The worker number skews to the king’s species (Fig 4), although there is a significant difference in worker numbers between mRa × fRa and mRa × fRs (Fig 4B), indicating that the queen’s species also influences worker numbers as the population grows. This study demonstrated that the sex of one parent can exert a greater influence on colony traits compared to the sex of the other parent.

Various factors can be considered as proximate causes for the parental sex-specific influence on colony traits, encompassing heritable elements (including sex-linked genetic factors, cytoplasmic factors, and transgenerational epigenetic factors) as well as socioenvironmental factors (such as parental behaviors and chemical communication). Parent-offspring communication, exemplified by trophallaxis, plays a pivotal role in determining soldier caste [28]. However, the parental sex-specific effects on soldier sex ratio cannot be solely explained by socioenvironmental influences from the parents. In addition, not only the soldier ratio but also the soldier sex ratio can vary depending on the colony’s growth stage (S1 Fig). Therefore, genetic factors alone cannot account for these results.

Phenotypic traits in offspring of various organisms can be influenced by heritable factors beyond DNA sequences [29, 30]. Several molecular mechanisms have been identified, including the transmission of genomic imprinting (DNA methylation and histone modification), small non-coding RNAs, and cytoplasmic factors like hormones and nutrients [31, 32]. All these elements are passed on through both the sperm and egg, although hormones and nutrients are exclusively inherited from the egg [33]. However, if the cytoplasmic substances were to control in vivo response mechanisms, the observed disparity in soldier sex ratio, despite a consistent proportion of soldiers in mRs × fRa, mRa × fRa, mRa × fRs, and mRs × fRs, would not be possible. This is because both sexes of offspring having the same cytoplasmic substance from the same maternal species. Instead, sex-specific epigenetic factors transmitted from parents might be implicated as immediate determinants of caste fate, as suggested by a mathematical model [9]. In *Reticulitermes* termites, it is known that epigenetic factors inherited from the king and queen can influence the caste determination of offspring (whether they follow the worker pathway or nymph pathway) [9, 34], and there is a potential for these epigenetic factors to also impact soldier differentiation. Interestingly, the reproductive system and soldier sex ratio are linked, as observed in species of the *Reticulitermes* genus. In the case of *R*. *speratus* and *R*. *virginicus*, which are AQS species [35, 36], the soldier sex ratio is biased towards females, while in non-AQS species, there is no sex ratio bias [17]. The identification of epigenetic factors responsible for the bias in soldier sex ratio may lead to a better understanding of the underlying reasons for this intriguing correlation. The current study does not directly investigate heritable factors, behaviors, and pheromones from the king and queen respectively; either or both factors could potentially be significant. Therefore, further research is required to clarify the proximate causes of sex-dependent parental effects on colony characteristics.

## Supporting information

S1 Text Supplementary materials and methods and results. (PDF)

S1 Fig Dynamics of caste and sex ratio in *R*. *speratus* colonies. (TIF)

S1 Dataset Data set for the results in the main text. (XLSX)

S2 Dataset Data set for the results in the S1 Text. (XLSX)
