# Supplementary material for: Maternal determination of soldier proportion and paternal determination of soldier sex ratio in hybrid Reticulitermes (Isoptera: Rhinotermitidae) termite colonies
Source: PLoS One. 2023 Nov 2;18(11):e0293096. doi: 10.1371/journal.pone.0293096 (PMC10621947; doi:10.1371/journal.pone.0293096)
Supplement: S1 Text — (PDF) [file pone.0293096.s001.pdf]

Supplementary Materials for

Maternal determination of soldier proportion and paternal determination of soldier sex ratio in hybrid *Reticulitermes* (Isoptera: Rhinotermitidae) termite colonies

This file includes:

S1 Text: Supplementary Materials and Methods and Results

S1 Fig

Other Supplementary Materials for this manuscript includes the following:

Dataset 1

Dataset 2

S1 Text

Supplementary Materials and Methods

Decayed logs containing *R. speratus* colonies were collected in pine or Japanese cedar forests in Kyoto, Japan, in April and May 2018 and 2022 (see Dataset 1 for details). The method of colony founding is the same as the one described in hybrid experiment section in the main text. See Dataset 2 for details of mating types and number of replications.

In colonies which were founded in 2022, all individuals from each colony were extracted at 0.5 years after colony foundation. In colonies which were founded in 2018, the extraction was conducted at 2.5 and 4.5 years after colony foundation. The weights of the kings and queens, as well as the weights of ten workers from each colony, were recorded. The numbers of eggs, larvae, workers, and nymphs were also recorded. For soldiers, they were sexed by sternite morphology and the counts and total weights for each sex was recorded.

Statistical analysis. Exact binomial tests were applied to compare the observed numerical soldier sex ratio in 1-, 2.5-, and 4.5-year-old colonies of *R. speratus* against the null hypothesis assuming that the numbers of males and females were equal. Comparison of the numerical soldier sex ratio or soldier ratio between 2.5- and 4.5-year-old colonies was performed with generalized linear models (GLMMs) with binomial distribution. In the models, the objective variable was the numerical sex ratio of soldiers (number of males vs females) or soldier ratio (number of soldiers vs workers), the explanatory variable was the age of the colonies, and colony ID was treated as a random factor. Comparison of the number of workers between 2.5- and 4.5-year-old colonies was performed with a GLMM with a Poisson distribution. In the model, the objective variable was the number of workers, the explanatory variable was the age of the colonies, and colony ID was treated as a random factor. For the GLMMs, a likelihood ratio test (LRT) was used to determine the statistical significance of the explanatory variable. A significance value of $p < 0.05$ was considered to indicate statistical significance.

Supplementary Results

Comparison of soldier sex ratio

There was no statistically significant bias in soldier sex ratio in the 1-year-old colonies (exact binomial test, 95% CI = 0.412–0.606, p = 0.924, S1 Fig). The sex ratio was significantly skewed toward females in 2.5-year-old colonies (exact binomial test, 95% CI = 0.694–0.770, $p < 0.001$, S1 Fig) and 4.5-year-old colonies (exact binomial test, 95% CI = 0.780–0.839, $p <$ 0.001). The soldier sex ratio in 4.5-year-old colonies was significantly more biased towards females than that in 2.5-year-old colonies (GLMM, LRT: $\chi^2 = 10.117$, $df = 1$, $p = 0.001$).

Comparison of soldier proportion

The soldier proportion in 4.5-year-old colonies was significantly lower than that in 2.5-year-old colonies (GLMM, LRT: $\chi^2 = 69.796$, $df = 1$, $p < 0.001$, S1 Fig).

Comparison of number of workers in a colony

There were significantly more workers in 4.5-year-old colonies than 2.5-year-old colonies (GLMM, LRT: $\chi^2 = 5756.9$, $df = 1$, $p < 0.001$, S1 Fig).

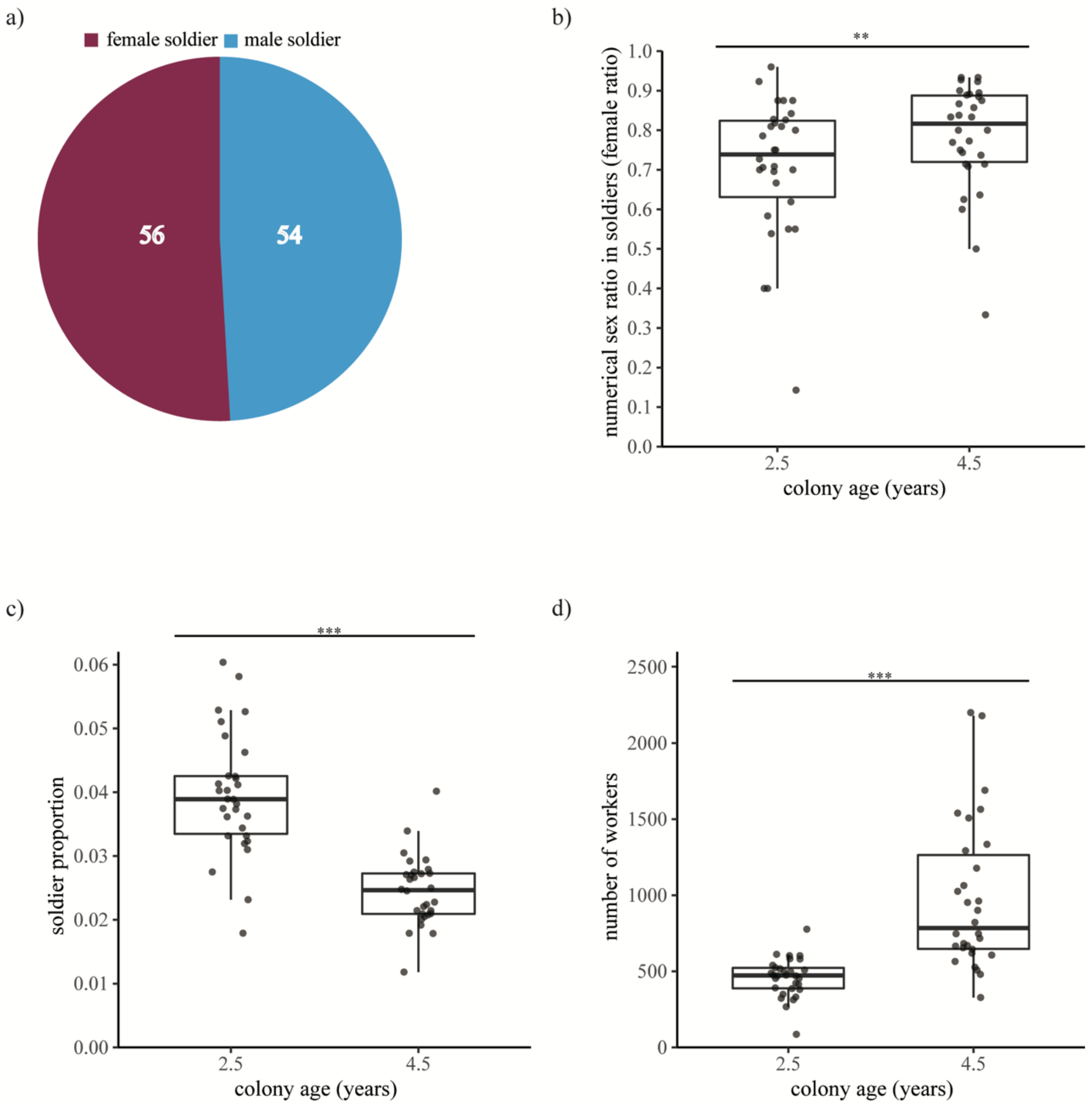

S1 Fig. Dynamics of caste and sex ratio in *R. speratus* colonies. a) No bias in sex ratio in the 0.5-year-old colonies. b), c), and d) Comparison of soldier sex ratio, soldier proportion, and number of workers between 2.5- and 4.5-year-old colonies, respectively. Asterisks indicate significant differences (likelihood ratio test, $^{**}p < 0.01$, $^{***}p < 0.001$).
