# Supplementary figures and images for: Maternal determination of soldier proportion and paternal determination of soldier sex ratio in hybrid Reticulitermes (Isoptera: Rhinotermitidae) termite colonies
Source: PLoS One. 2023 Nov 2;18(11):e0293096. doi: 10.1371/journal.pone.0293096 (PMC10621947; doi:10.1371/journal.pone.0293096)

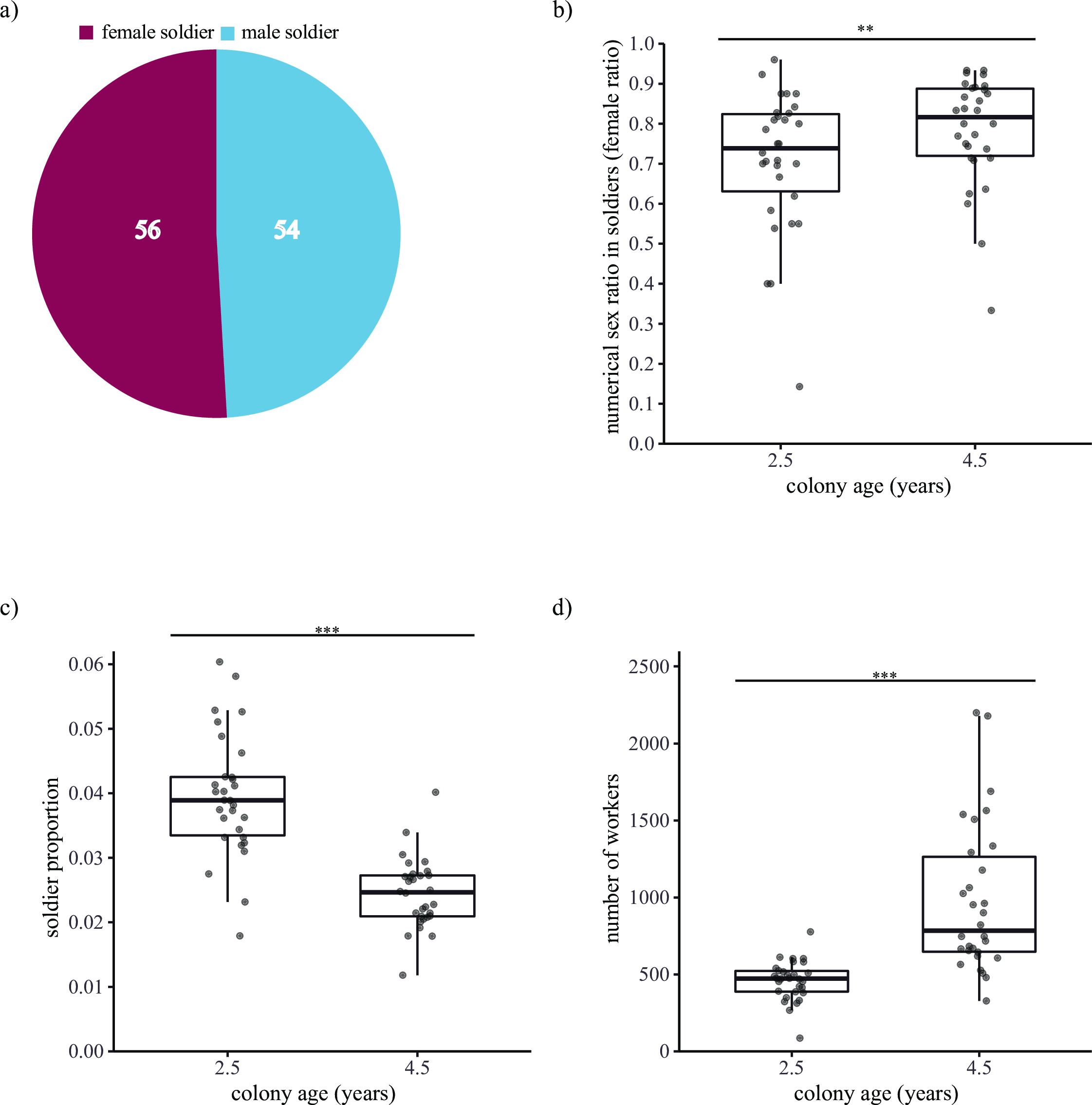

Supplement: S1 Fig — (TIF) [file pone.0293096.s002.tif]
